# CXCR2-Blocking Has Context-Sensitive Effects on Rat Glioblastoma Cell Line Outgrowth (S635) in an Organotypic Rat Brain Slice Culture Depending on Microglia-Depletion (PLX5622) and Dexamethasone Treatment

**DOI:** 10.3390/ijms242316803

**Published:** 2023-11-27

**Authors:** Johannes Falter, Annette Lohmeier, Petra Eberl, Eva-Maria Stoerr, Janne Koskimäki, Lena Falter, Jakob Rossmann, Tobias Mederer, Nils Ole Schmidt, Martin Proescholdt

**Affiliations:** 1 Department of Neurosurgery, University Hospital Regensburg, 93042 Regensburg, Germany; 2 Department of Neurosurgery, Oulu University Hospital, P.O. Box 25, 90029 Oulu, Finland; 3 Department of Anesthesiology, Caritas Hospital St. Josef Regensburg, 93053 Regensburg, Germany

**Keywords:** glioblastoma, OBSC, dexamethasone, PLX5622, CXCL2, danirixin, navarixin, microglia

## Abstract

In glioblastoma (GBM), the interplay of different immune cell subtypes, cytokines, and/or drugs shows high context-dependencies. Interrelations between the routinely applied dexamethasone (Dex) and microglia remain elusive. Here, we exploited rat organotypic brain slice co-cultures (OBSC) to examine the effects on a rat GBM cell line (S635) outgrowth resulting from the presence of Dex and pretreatment with the colony-stimulating factor receptor 1 (CSF1-R) inhibitor PLX5622: in native OBSC (without PLX5622-pretreatment), a diminished S635 spheroid outgrowth was observable, whereas Dex-treatment enhanced outgrowth in this condition compared to PLX5622-pretreated OBSC. Screening the supernatants of our model with a proteome profiler, we found that CXCL2 was differentially secreted in a Dex- and PLX5622-dependent fashion. To analyze causal interrelations, we interrupted the CXCL2/CXCR2-axis: in the native OBSC condition, CXCR2-blocking resulted in increased outgrowth, in combination with Dex, we found potentiated outgrowth. No effect was found in the PLX5622-pretreated. Our method allowed us to study the influence of three different factors—dexamethasone, PLX5622, and CXCL2—in a well-controlled, simplified, and straight-forward mechanistic manner, and at the same time in a more realistic ex vivo scenario compared to in vitro studies. In our model, we showed a GBM outgrowth enhancing synergism between CXCR2-blocking and Dex-treatment in the native condition, which was levelled by PLX5622-pretreatment.

## 1. Introduction

Glioblastoma (GBM) is still a fatal diagnosis with excessive infiltration of tumor cells into the brain parenchyma, early neurological deficits, and inevitable recurrence despite maximum therapy [1].

GBM-associated edema causes high symptom burden, which is commonly treated with dexamethasone (Dex) for symptom control [2]. For dexamethasone treatment, contradictory effects have been described in numerous in vitro, in vivo, or clinical studies, either promoting or hampering GBM aggressiveness. Earlier studies, and especially results from in vitro investigations, attributed direct anti-tumoral effects to dexamethasone treatment [3,4,5,6,7]. These results were sustainably challenged by clinical data, indicating that dexamethasone mediates impediments of GBM therapy or may even cause worse overall survival [8,9,10,11].

It is well known that dexamethasone has general suppressive effects on the immune system. In particular, with the emergence of immunotherapies and other immune-related therapies (e.g., oncolytic viruses) [12,13,14,15], the old debate about the benefits of dexamethasone versus the dexamethasone-induced detrimental immunosuppressive effects experienced a renaissance [16,17,18,19].

Within the immunological tumor microenvironment (TME), microglia are the most numerous immune cell type in newly diagnosed GBM [20]. The microglial compartment is part of the so-called GAMM (GBM-associated microglia and macrophages), which poses context-dependent starting points for therapeutic interventions [21]. High numbers of GAMM have been correlated with worse overall survival, but GAMM reprogramming or even depletion showed preclinical benefits in certain studies but did not prove effective in clinical trials [22]. 

Higher fractions of microglial cells in the immune cell compartment were correlated with enhanced overall survival in GBM patients [23]. Even though brain-resident microglia and bone-marrow-derived GBM-invading macrophages differ ontogenetically and functionally enormously, differentiation between these GAMM subsets is still challenging [24,25,26].

As microglia comprise the majority of the so-called GAMM in newly diagnosed glioblastoma [27], comprehending the effects of the commonly used dexamethasone on the microglial response is relevant for understanding underlying mechanisms in primary GBM. It may even lead to approaches explaining the failure of GAMM- or microglia-modulating therapies [28]. Organotypic rat brain slice co-cultures (OBSC) offer the unique opportunity to observe the microglial immune compartment isolated from macrophages, as recruitment of other immune cells from the periphery is impossible, and the invasion of bone-marrow-derived macrophages cannot occur in that model.

The crosstalk between GBM cells and GAMMs is influenced by CXCL2-CXCR2 signaling [29,30,31]. Overexpression of CXCL2 in GBM is correlated with worse overall survival and tumor malignancy [29,32,33]. In the literature, blocking of the CXCL2-receptor (CXCR2) mediated a reduced tumor outgrowth [33], and decreased tumor volumes, at least preclinically in orthotopic GBM mouse models [32,34,35].

In this study, we investigate the interrelations between microglia (and PLX5622-pretreatment), the CXCL2-CXCR2 axis, and dexamethasone treatment in a very well-controlled setting in order to examine the context-dependencies of this ménage à trois. We try to balance between mechanistic simplification and the rather close-to-reality ex vivo scenario of the rat brain slice cultures.

## 2. Results

### 2.1. Effects of PLX5622-Pretreatment and Dexamethasone Treatment on S635 GBM Rat Cell Line Outgrowth

The rat glioblastoma cell line S635 and control astrocytes underwent vital fluorescence staining before they were applied as a cell spheroid-like conglomerate on hippocampal rat organotypic brain slice co-cultures (OBSC), which were pretreated with the CSF1-Receptor antagonist PLX5622 (for microglia-depletion) or were treated with PBS as controls before (native OBSC condition) [36]. PLX5622 is a microglia-depleting agent that causes a very specific microglial depletion with minor influences on other cells on the OBSC [37]. Spheroid outgrowth was determined with fluorescent area measurement via digital intensity thresholding within the rat OBSC after seven days. 

**Figure 1:**
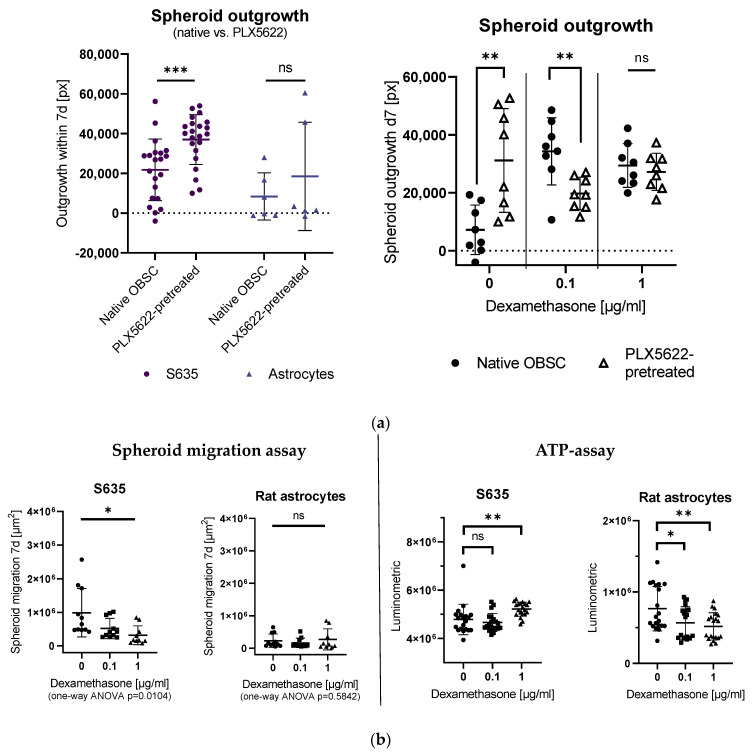
(**a**) Left: Comparison of spheroid outgrowth for S635 and rat astrocytes in the native organotypic brain slice cultures (OBSC) vs. PLX5622-pretreated conditions without any other treatment. Spheroid outgrowth was significantly reduced in the native OBSC condition in the S635 cell line (unpaired *t*-test, *p* = 0.0010). No difference was detectable in the astrocyte control cell line in both conditions (unpaired *t*-test, *p* = 0.4242). Right: In our rat OBSC, dexamethasone (Dex)-treatment led to a significantly enhanced outgrowth in the 0.1 µg/mL Dex-treated condition (two-way ANOVA, effect for Dex-treatment *p* = 0.0378), whereas in the PLX5622-pretreated condition, a rather tumor outgrowth-inhibiting effect of Dex was detectable (two-way ANOVA, interaction pretreatment/Dex *p* < 0.0001). In pairwise comparisons, there was a significantly enhanced outgrowth at baseline for the PLX5622-pretreated group (unpaired *t*-test, *p* = 0.0042), but with 0.1 µg/mL dexamethasone treatment, we found significantly less outgrowth on the PLX5622-pretreated OBSCs (unpaired *t*-test, *p* = 0.0064) (**b**) Left: Spheroid migration on plastic was significantly reduced in the Dex-treated S635 (one-way ANOVA, *p* = 0.0104) cell line in a concentration-dependent manner. No effect was seen for the generally low astrocytic migration (one-way ANOVA, *p* = 0.5842). Right: In an ATP assay, no significant difference was found for S635 between the control and 0.1 µg/mL Dex concentration (unpaired *t*-test, *p* = 0.4567); cell death was even reduced with 1 µg/mL dexamethasone (unpaired *t*-test, *p* = 0.0087). The rat astrocyte cell line showed Dex concentration-dependent cell death in vitro (unpaired *t*-test, *p* = 0.0268 and *p* = 0.0044). Significance is marked with * *p* < 0.05; ** *p* < 0.01; *** *p* < 0.001; “ns” implies a non-significant *p* value.

Outgrowth was significantly enhanced in the PLX5622-pretreated OBSC condition for the S635 cell line, whereas for the astrocyte control cell line, no difference was detectable between the two conditions (see Figure 1a, left). 

In our rat OBSC, dexamethasone treatment also led to a significantly enhanced S635 outgrowth in the native OBSC condition when 0.1 µg/mL dexamethasone was applied, whereas in the PLX5622-pretreated condition, an outgrowth-inhibiting effect of dexamethasone was detectable. The excessive concentration of 1 µg/mL dexamethasone leveled these differences between the two OBSC conditions (see Figure 1a, right).

To investigate the direct effect of dexamethasone on the isolated rat GBM cells in vitro, we first conducted an in vitro spheroid migration assay with different concentrations of dexamethasone (0.1 µg/mL and 1 µg/mL), measuring the increase of the cell-covered area within seven days. Spheroid migration on plastic was significantly reduced in the dexamethasone-treated S635 cell line conditions in a concentration-dependent manner (see Figure 1b, left). 

To check dexamethasone-induced cell death in our cell lines, we applied an ATP assay. We found no difference between control and 0.1 µg/mL dexamethasone treatment, but enhanced cell viability for S635 in the excessive dexamethasone concentration (1 µg/mL). The rat astrocyte control cell line showed decreased cell viability with increasing dexamethasone concentrations in vitro (see Figure 1b, right). 

### 2.2. Influence of PLX5622-Pretreatment and Dexamethasone on CXCL2 Secretion

**Figure 2:**
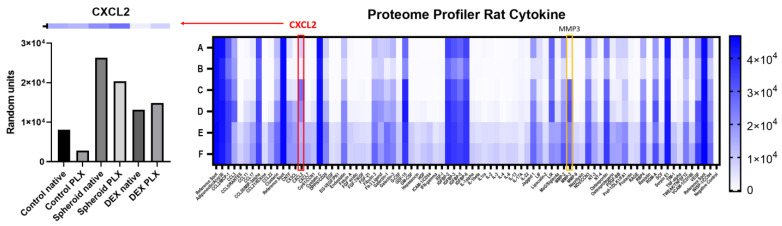
Heat map of the membrane-based proteome profiler (XL Rat cytokine, R&D systems): A (control) = native OBSC without spheroid; B (control) = PLX5622-pretreated OBSC without spheroid; C = native OBSC with spheroid; D = PLX5622-pretreated OBSC with spheroid; E = native OBSC with spheroid + Dex treatment; F = PLX5622-pretreated OBSC with spheroid + Dex treatment; Pooled samples from supernatants from two timepoints and four different replicate wells. Notably, MMP3 shows a conclusive signal coming from the presence of the spheroid (marked with yellow box). CXCL2 (marked with red box) shows spheroid- (almost none), PLX5622-pretreatement- (highest in native OBSC), and Dex-dependent (medium) concentrations, which indicates a potentially important role of the CXCL2-axis in spheroid outgrowth within our model. On the left, the values for CXCL2 are presented in graph bars for each condition (A–F).

To screen for relevant cytokine signaling in the two conditions (native OBSC vs. PLX5622-pretreated) in combination with dexamethasone application, we pooled supernatants from two time points and four different replicates of each condition and screened with a semi-quantitative, membrane-based proteome profiler (R&D systems). We included OBSC without the GBM cell spheroid in our analysis (see Figure 2, row A/B) in the native OBSC (A) and PLX5622-pretreated (B) condition, which delineates the cytokine signaling from an isolated brain slice without the GBM spheroid as a baseline.

We could observe a general background from the serum, supplements, and other culture medium constituents but were still able to detect differential signaling: e.g., for MMP-3, we saw no signal in the control OBSCs without the GBM spheroid, but we could observe a good signal in the conditions with the GBM spheroid, indicating that the screening produced conclusive results for certain differential cytokine concentrations, despite the background (see Figure 2).

Comparing the respective conditions with each other, we found a spheroid-, PLX5622-pretreatment-, and dexamethasone-dependent secretion for CXCL2 and a >0.75-fold higher signal in the native OBSC condition with spheroid present compared to the native OBSC condition without spheroid, which indicated a potentially important role in the interaction of the spheroid with the TME in our model, so that we further investigated and quantified CXCL2 in our model.

To further investigate our semi-quantitative observation, we performed ELISA to quantitatively check for the CXCL2 concentrations in the supernatants per well and timepoint (day 0 = baseline after PLX5622-treatment, day 3 and day 6). 

**Figure 3:**
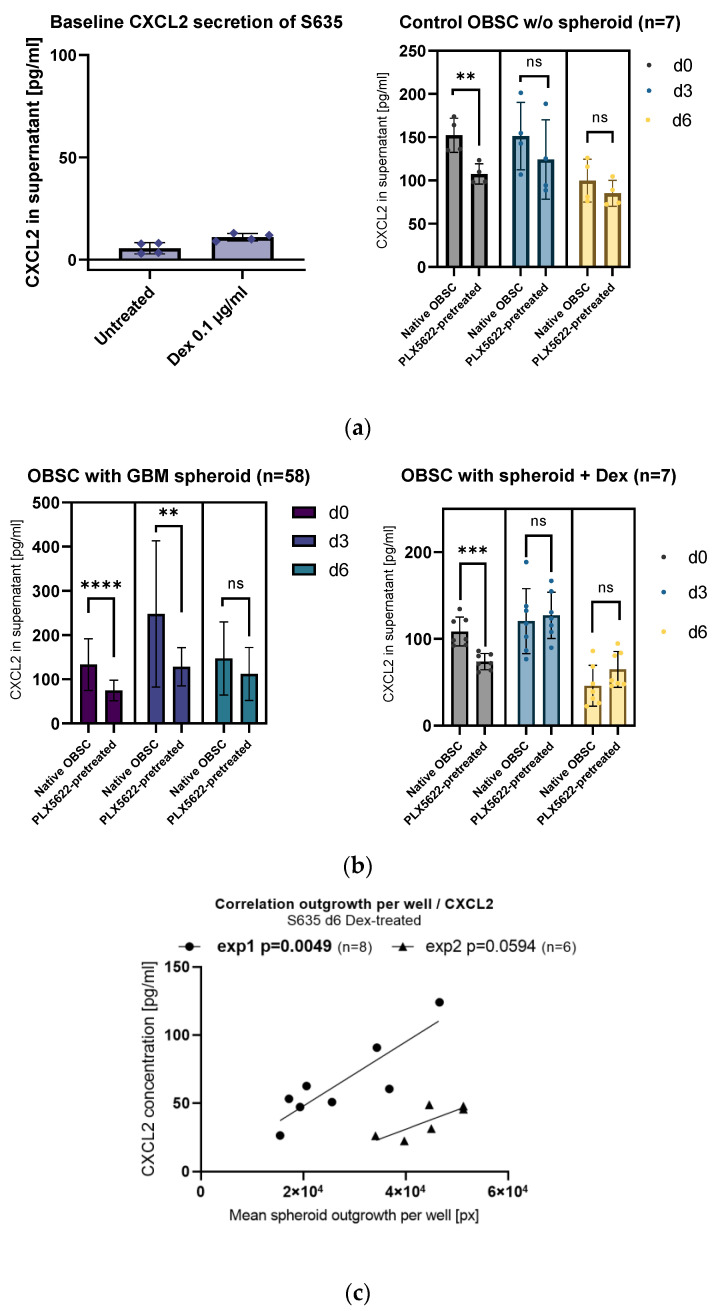
(**a**) Left: Isolated S635 cell line cells show very low baseline of CXCL2 in the supernatants. Right: In the control OBSC (without spheroid/without treatment), the observed difference between native and PLX5622-pretreated from day 0 (unpaired *t*-test, *p* = 0.0004) levels out from day 3 (unpaired *t*-test, *p* = 0.7085) and day 6 (unpaired *t*-test, *p* = 0.1376). (**b**) Left: Generally, the PLX5622-pretreatment results in less CXCL2 secretion (two-way ANOVA, effect for treatment *p* < 0.0001). Looking at all the OBSCs with the spheroid present, CXCL2 secretion peaks in the native OBSC condition at day 3 and significantly goes down at day 6 (two-way ANOVA, effect for timepoint *p* = 0.0016). Pairwise comparisons show a significantly diminished CXCL2 concentration in PLX5622-pretreated OBSC supernatants at d0 (unpaired *t*-test, *p* < 0.0001) and at d3 (unpaired *t*-test, *p* = 0.0018), whereas the difference is leveled at d6 (unpaired *t*-test, *p* = 0.7636). Right: CXCL2 secretion with Dex is by trend higher in the PLX5622-pretreated condition. At day 6, the general CXCL2 concentration in the supernatant is very low. We see a significant difference at d0 between native and PLX5622-pretreated OBSC (unpaired *t*-test, *p* = 0.0004), but the difference levels at day 3 (unpaired *t*-test, *p* = 0.7085) and day 6 (unpaired *t*-test, *p* = 0.1376). (**c**) A direct correlation of CXCL2 secretion in the supernatant per well and spheroid outgrowth per well only showed in the group with Dex treatment. Significance is marked with ** *p* < 0.01; *** *p* < 0.001; **** *p* < 0.0001; “ns” implies a non-significant *p* value.

In order to check if a significant proportion of the CXCL2 concentration in the supernatants comes from baseline CXCL2 secretion from the cell line, we cultured isolated GBM cell line spheroids (4000 cells per spheroid) on plastic. After culturing the spheroid for 7 days in one-tenth of the volume compared to the volume of culture medium of the OBSC, we still found a very low baseline CXCL2 secretion in the supernatant (up to 13 ng/mL), indicating a negligible baseline CXCL2 secretion from the cell line itself (see Figure 3a, left).

Baseline CXCL2 secretion coming from the brain slice cells (OBSC without the GBM spheroid) was generally lower (see Figure 3a, right). In the untreated d0 supernatants, there was an overall significant difference between the native OBSC condition and the PLX5622-pretreated condition (see Figure 3a, right). However, in the supernatants from day 3 and day 6, the difference between the two conditions (native OBSC vs. PLX5622-pretreated) was leveled (see Figure 3a, right). 

Looking at all the CXCL2 concentrations on d3 supernatants regardless of their treatment, we found a highly significant difference between the two OBSC conditions (native OBSC vs. PLX5622-pretreated), showing that the PLX5622-pretreatment causes a general reduction in CXCL2 signaling, but only when the spheroid is present on the OBSC (see Figure 3b, left). On day 6, the difference between the two conditions is leveled. 

We could also observe a more pronounced CXCL2 signaling after placement of the spheroid and less CXCL2 signaling in our OBSC model in the second half of the observations period (supernatant at timepoint d6), which might be due to the GBM cell line cells starting to suppress CXCL2-CXCR2 axis signaling over time (see Figure 3b, left). 

With dexamethasone treatment, the spike of secretion of CXCL2 at day 3 after spheroid placement is completely missing. With dexamethasone treatment, the CXCL2 secretion is significantly suppressed over time (see Figure 3b, right). 

Interestingly, with dexamethasone treatment, the very low CXCL2 concentrations show a direct correlation with GBM cell line outgrowth on PLX5622-pretreated rat OBSCs (see Figure 3c).

### 2.3. Context-Sensitive Impact of CXCL2-Receptor (CXCR2)-Blocking on Spheroid Outgrowth

To further investigate the role of the CXCL2/CXCR2-axis in our model, we blocked the corresponding CXCL2-receptor (CXCR2) with two different inhibitors (small molecules danirixin and navarixin) and via non-directional overstimulation with recombinant CXCL2.

**Figure 4:**
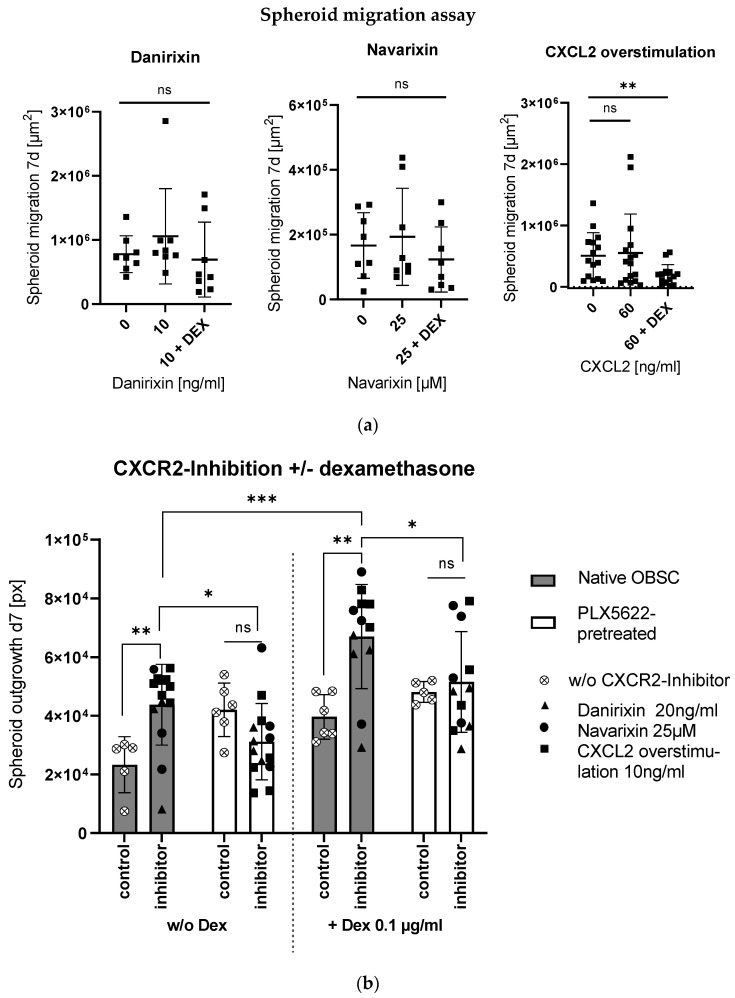
(**a**) CXCR2-inhibition showed no significant effect on isolated cell migration in the spheroid migration assay. For CXCL2 overstimulation in combination with Dex, we see a significant reduction of spheroid migration (unpaired *t*-test, *p* = 0.0082). (**b**) Bar graph shows spheroid outgrowth after 7 days depending on pretreatment (native/PLX5622-pretreated) and treatment without and with CXCR2-inhibitors. Moreover, combinations without and with Dex (left and right side) are displayed. Significances in the pairwise comparisons are marked. CXCR2-inhibition led to enhanced outgrowth in the native OBSC condition (unpaired *t*-test, *p* = 0.0074), which was even potentiated in combination with Dex (unpaired *t*-test, *p* = 0.0010). In the PLX5622-pretreated condition, there was no effect with the CXCR2-inhibitor treatment without (unpaired *t*-test, *p* = 0.0807) nor in combination with Dex (unpaired *t*-test, *p* = 0.6692). Reduced outgrowth was found in PLX5622-pretreated OBSC treated without and with Dex in combination with CXCR2-inhibitors compared to the native OBSC condition (unpaired *t*-test, *p* = 0.0196 and *p* = 0.0413). Significance is marked with * *p* < 0.01; ** *p* < 0.01; *** *p* < 0.001; “ns” implies a non-significant *p* value.

To check the direct effects of the inhibitory agents on the isolated GBM cell line S635, a spheroid migration assay was performed, where the small molecules danirixin and navarixin and the blocking non-directional CXCR2-overstimulation showed no effect on migration; in combination with dexamethasone, less migration was seen in the condition with CXCL2 overstimulating treatment (see Figure 4a). 

In our ex vivo OBSC model, the three inhibitory conditions induced enhanced outgrowth in the microglia-native condition, which was even potentiated and, therefore, produced a tumor-promoting effect in combination with dexamethasone (see Figure 4b). In the microglia-depleted condition, no changes in outgrowth with CXCR2-inhibition were observed, or in combination with dexamethasone (see Figure 4b). 

### 2.4. Cellular Correlate (Microglia [Iba1^+^] and Astrocytes [GFAP^+^]) within the Rat OBSC

As rat OBSCs slices were archived at −80 °C after the experiments, we were able to cryosection the rat OBSCs and immunofluorescence stain for specific cells (Iba1^+^ for microglia and glial fibrillary acidic protein [GFAP^+^] for astrocytes).

**Figure 5:**
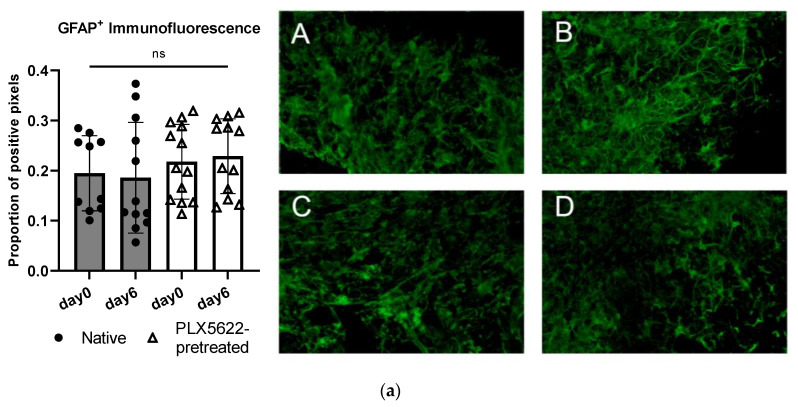

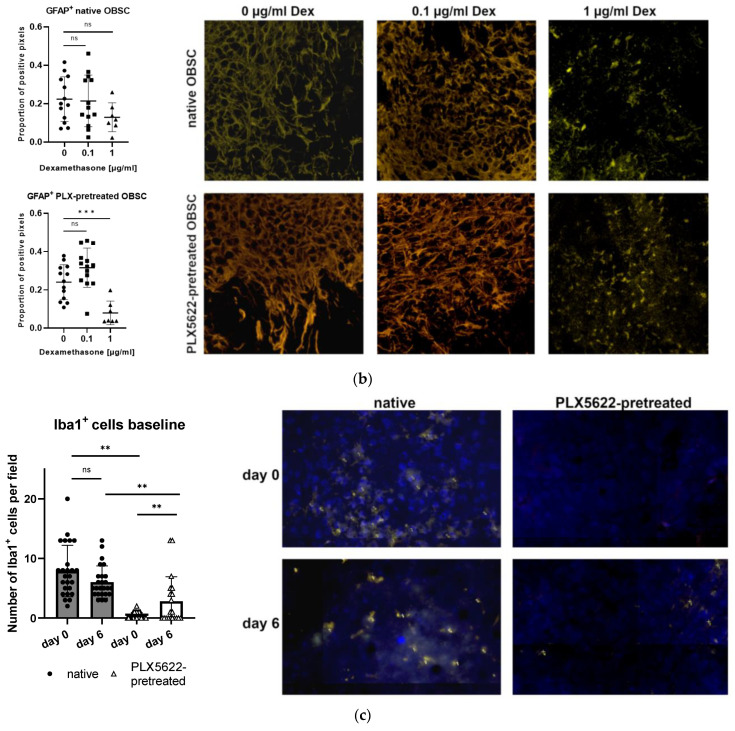

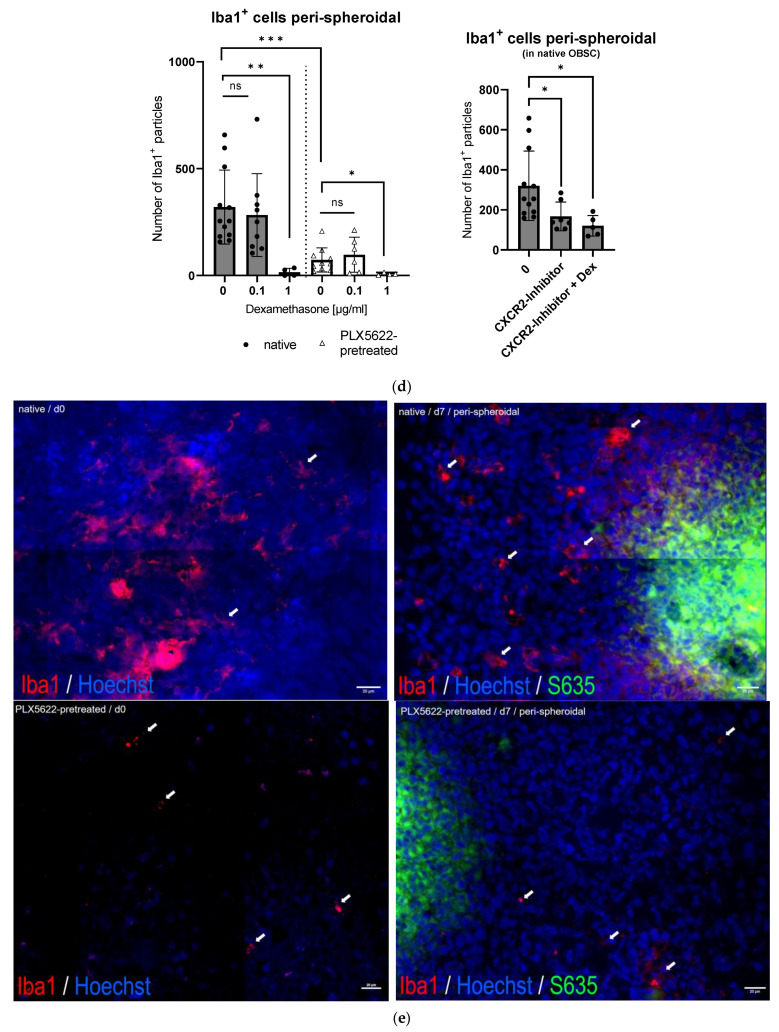
(**a**) Quantification of glial fibrillary acidic protein (GFAP) in control tissue sections without spheroid (native vs. PLX5622 pretreated) on day 0 (first day after pretreatment) and on day 6 without any other treatment. There is no significant change between the two time points (d0 and d6) and between the groups (one-way ANOVA, *p* = 0.5891). Representative images of immunofluorescently stained (green: GFAP^+^) sections A = day0/native, B = day0/PLX5622-pretreated, C = day6/native, D = day6/PLX5622-pretreated. (**b**) Left: Proportion of GFAP^+^ pixels in native OBSC with spheroid (upper) and PLX5622-pretreated OBSC on day 6 (lower) after Dex treatment. No significant difference between Dex untreated, 0.1 µg/mL, and 1 µg/mL Dex-treated slices (unpaired *t*-test, *p* = 0.8531 and *p* = 0.0695) was detectable. In PLX5622-pretreated OBSC, there was no significant difference between Dex untreated OBSC and 0.1 µg/mL treated slices (unpaired *t*-test, *p* = 0.0537), whereas significantly diminished GFAP positivity was seen for 1 µg/mL Dex-treated slices (unpaired *t*-test, *p* < 0.0006). Right: Representative images of GFAP-stained sections (yellow: GFAP^+^). Only the excessive Dex concentration of 1 µg/mL leads to significant astroglial degeneration. (**c**) For Iba1-staining (blue: Hoechst33342, yellow: Iba1^+^), there is no difference in Iba1^+^ cells in the native condition between day 0 and day 6 (*p* = 0.0769, unpaired *t*-test), but significantly fewer Iba1^+^ cells in the PLX5622-pretreated condition compared to the native condition on day 0 (*p* < 0.0001) and day 6 (*p* = 0.0054) was found. We see a significant regeneration of microglia within six days in the PLX5622-pretreated group (*p* = 0.0028), but compared to the native condition, there are still significantly less Iba1+ cells in PLX5622-pretreated OBSCs. (**d**) Left: In the peri-spheroidal area at day 7, we find a significant effect for Dex treatment and the native/PLX5622-pretreatment condition (two-way ANOVA, no interaction, *p* = 0.1096; effect for Dex treatment, *p* = 0.0048; effect for pretreatment native/PLX5622; *p* = 0.0017). Pairwise comparisons show no significant difference between 0 µg/mL and 0.1 µg/mL Dex in both conditions (unpaired *t*-test, *p* = 0.6456 [native], and *p* = 0.4917 [PLX5622]), whereas significantly less Iba1^+^ particles can be detected comparing 0 µg/mL and 1 µg/mL Dex conditions in native and PLX5622 pre-treated slices (unpaired *t*-test, *p* = 0.0041 [native], and *p* = 0.0424 [PLX5622]). Comparing native and PLX5622-pretreated OBSC without Dex treatment, we find significantly less Iba1^+^ particles peri-spheroidally in PLX5622-pretreated OBSC (unpaired *t*-test, *p* = 0.0003). Right: We found a significant reduction in Iba1^+^ cells in the peri-spheroidal area with CXCR2-Inhibitor (unpaired *t*-test, *p* = 0.0408) and after treatment with CXCR2-Inhibitor and Dex (0.1 µg/mL) (unpaired *t*-test, *p* = 0.0247). (**e**) Representative images showing morphological features of our Iba1^+^-stained cells (microglial cells are marked with white arrows, scale bar indicates 20 µm; red: Iba1^+^, blue: Hoechst33342, green: S635). We could find ramified microglia in d0 untreated native OBSC (upper left), amoeboid (=activated) microglial features at d7 in native OBSC in the peri-spheroidal area (upper right). Dystrophic Iba1^+^ microglia can be found on day 0 (lower left) and amoeboid—but scarce microglia at d7 peri-spheroidally in PLX5622-pretreated OBSC (lower right). Significance is marked with * *p* < 0.01; ** *p* < 0.01; *** *p* < 0.001; “ns” implies a non-significant *p* value.

**Figure 6:**
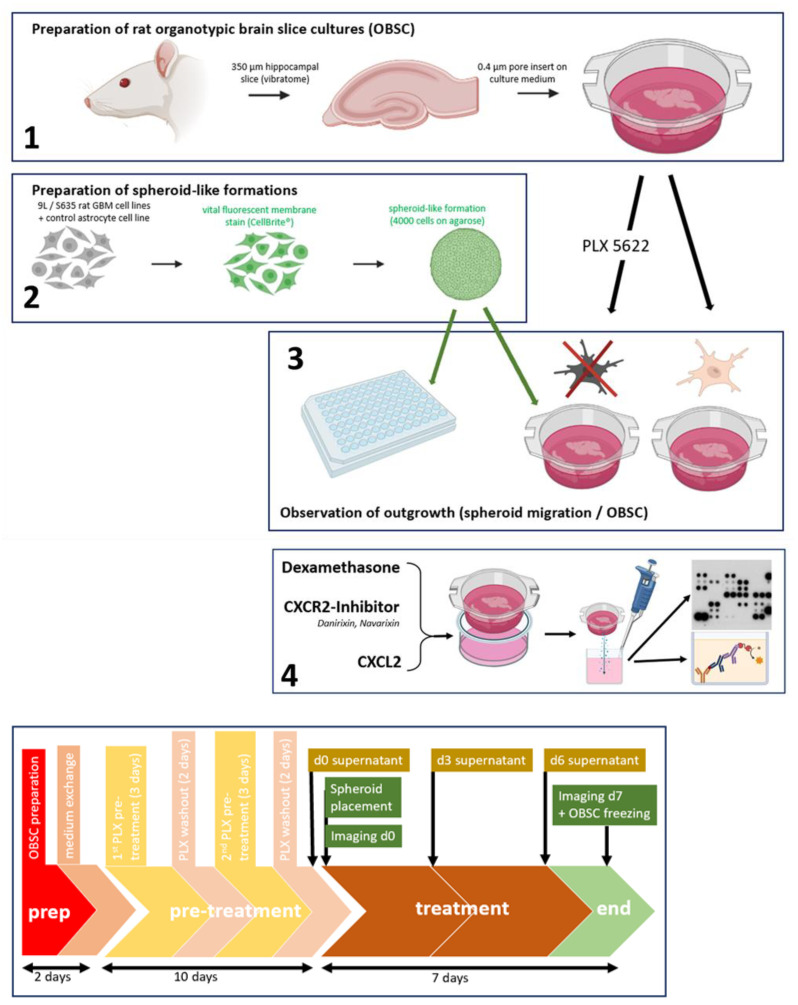
Brains from d6–d12 rats were cut into 350 µm slices and put on special 0.4 µm pore inserts floating on culture medium (1). S635 and a control rat astrocyte cell line were vitally stained (CellBrite green), conglomerated into 4000-cell spheroid-like structures on agarose (2) and then transferred onto the rat OBSC or on a 96 U-well plate with a pipette tip. Outgrowth was determined after 7 days by measuring the increase of the fluorescent area. We conducted PLX5622-pretreatment (2 × 3 days) of the OBSC (3). DEX and/or CXCL2 or CXCR2-inhibitors were added to the medium for different treatment conditions. A membrane-based semi-quantitative proteome profiler was used to screen for differential cytokine signaling in the supernatants. ELISA was performed to quantify the CXCL2 concentrations in the supernatants (4). Bottom: summarized chronological sequence of the experiments (illustrations were generated with biorender.com (accessed on 30 June 2023).

In the astroglial compartment of the OBSC, we neither found a difference in GFAP^+^ -immunofluorescence signal between the native and the PLX5622-pretreated condition, nor did we detect a difference between the two different timepoints on baseline OBSC without spheroid (see Figure 5a). Day 0 marks the timepoint before the spheroid is usually applied, which is directly after the pretreatment period; day 6 marks the timepoint after the treatment period, one day before outgrowth was usually documented (see Figure 5a and Figure 6). 

For the dexamethasone treatment, no difference in GFAP^+^-immunofluorescence was found in both conditions (native and PLX5622-pretreated) between 0 µg/mL dexamethasone and 0.1 µg/mL dexamethasone treatment. The excessive concentration of 1 µg/mL, though, led to a significantly diminished GFAP-fluorescence signal in PLX5622-pretreated slices (see Figure 5b).

Immunofluorescent Iba1^+^ staining was performed on the baseline control slices (without spheroid) to control the success of the microglia-depletion on the OBSC at the two-time points (d0, d6). On day 0 and day 6, significantly less Iba1^+^ cells were found in the PLX5622-pretreated condition, whereas over time, comparing day 0 and day 6, a certain regeneration of Iba1^+^ cells can be found with a significantly higher Iba1^+^ cell content on day 6 in the PLX5622-pretreated condition compared to PLX5622-pretreated OBSC on d0. But, a significant difference between the “microglia-depleted” condition (PLX5622-pretreated) and the native OBSC is preserved until day 6 (see Figure 5c).

In the peri-spheroidal area, the number of Iba1^+^ cells differs significantly, depending on the pretreatment (see Figure 5e). Dexamethasone treatment with 0.1 µg/did not lead to a reduction of Iba1^+^ signal, but treatment with 1 µg/mL dexamethasone resulted in a significantly diminished Iba1^+^ signal in both conditions (native and PLX5622-pretreated) (see Figure 5d, left). 

Treatment with CXCR2-inhibiting agents also led to fewer Iba1^+^ particles in the peri-spheroidal area of native OBSC; in combination with dexamethasone, this decrease is even more pronounced (see Figure 5d, right). 

Morphologically, in native OBSC without spheroid on day 0 (10 days of sham pretreatment), we found ramified/resting microglia. In the peri-spheroidal area, on day 6, we found abundant activated amoeboid microglia (see Figure 5e). Despite depletion, we could also find amoeboid microglia in the peri-spheroidal area of PLX5622-pretreated conditions at day 7, but very scarcely. Directly after pretreatment with the CSF1-R-antagonist, depleting agent PLX5622, only dystrophic microglia with a very low Iba1^+^ signal is distinguishable (see Figure 5e).

## 3. Discussion

Here, we were able to evaluate the impact on spheroid outgrowth of two different types of OBSC spheroid microenvironments, microglia-native or microglia-depleted (PLX5622-pretreated), and the context-dependent effects of different treatments (dexamethasone, CXCR2-inhibition). 

PLX5622-pretreatment depleted microglia without affecting the astroglial compartment. Even though a certain regeneration of microglia over time occurred in our setting and has previously been described [38], microglia-depletion with PLX5622 led to a sustainably lowered microglia cell number until the end of our experiment. 

Compared to the microglia-depleted (PLX5622-pretreated) condition, we saw reduced spheroid outgrowth on the native OBSC. 

It has been shown that microglia can act as immunological effector cells [39] setting limits to spheroid outgrowth. Morphologically, we found activated amoeboid microglia peri-spheroidally in our OBSC, underscoring the activation and anti-tumoral activity of this cell compartment. 

At least to a certain extent, placement of the spheroid onto the OBSC mimics the early microglial reaction in the peritumoral zone, where the microglial compartment constitutes the most significant immune cell compartment, when macrophage invasion has not yet occurred [40,41]. Detachment of the brain slice from the systemic circulation enabled us to examine the influence of the isolated microglial compartment without macrophages infiltrating from the periphery. Thus, we could evade the difficulty of differentiating between the separate GAMM subgroups (microglia/macrophages) [26] and consequently observe the isolated microglial effect. 

Depending on the composition of our spheroidal microenvironment, dexamethasone led to enhanced outgrowth on native OBSC but reduced outgrowth in PLX5622-pretreated (microglia-depleted) OBSC. 

The effect of dexamethasone on survival and outcome, and its role in GBM treatment is still controversial [13,42,43]. Dexamethasone inhibited migration of our cell line cells on plastic. This reflects the variety of previous in vitro research that showed direct anti-GBM-cell line effects, attributing even anti-tumoral effects to dexamethasone treatment [3,4,5,6,7]. 

In our ex vivo OBSC model, though, dexamethasone treatment resulted in enhanced spheroid outgrowth, which either is mediated by the native tumor microenvironment, in particular through microglia, or is due to the immunosuppressive/microglia suppressive effect of dexamethasone. The pro-tumoral interaction of dexamethasone with the native OBSC microenvironment is in line with previous observations indicating that dexamethasone induces a specific form of dysfunctional microglia [44].

Signaling originating from GAMM has been shown to have either pro-tumorigenic or anti-tumorigenic effects [45,46], and context-dependent symbioses have been described [25]. 

The role of immune modulatory cytokines released from microglia [47] also seems to be, at least partially, the reason for the dexamethasone-dependent outgrowth-inhibiting or promoting effect.

Here, we identified CXCL2 as a differential cytokine with secretion depending on the presence of the cell line spheroid, dexamethasone treatment, and/or PLX5622 pretreatment.

CXCL2 is described to be secreted mainly by astrocytes in the brain, but also from macrophagic cell types, whereas the corresponding receptor CXCR2 is expressed by microglia and other immune cells [48]. Even though the exact CXCL2-secreting cell type remains unclear in our setting, changes in CXCL2 secretion and the general humoral fingerprint depending on (pre-)treatments were analyzed. From the evaluation of the CXCL2 concentrations in our controls, we can conclude that the secretion only from the isolated OBSC rat brain cells or from the isolated GBM spheroid is not as high as the combination of the GBM spheroid and OBSC, i.e., interaction of the spheroid with the co-cultured cells from the OBSC leads to enhanced CXCL2-cytokine secretion. In general, at day 0 (no treatment), directly after PLX5622 washout, the difference in CXCL2 concentrations between the native OBSC condition and PLX5622-pretreated condition is most pronounced, possibly due to reversible alterations of CXCL2-secreting rat brain cell types (e.g., astrocytes) of the TME through CSF1-receptor antagonism of PLX5622 and microglial depletion. These alterations seem to be reversible, as the difference between the two groups in the control OBSC without spheroid levels over time, indicating that the basal CXCL2 secretion without spheroid stimulus does not differ between the native OBSC condition and PLX5622-pretreated condition. 

Generally, secretion of CXCL2 decreases over time within the seven days of our observation period. Within the first three days, secretion is much higher, which might reflect a first tumor microenvironmental reaction to the GBM cell line spheroid. In the second half of our observation period, the CXCL2 secretion decreases, possibly showing a re-educational effect on the tumor microenvironment coming from the spheroid’s interaction with the rat brain slice cells. 

In line with clinical observations of CXCL2 concentration directly correlating with worse overall survival [29,32,33], we could find a correlation between spheroid outgrowth and CXCL2 concentrations in the supernatants, but only in the dexamethasone-treated group, which might even reflect a disadvantageous effect of the ubiquitously applied symptomatic dexamethasone treatment of GBM in the clinical practice. 

Blockage of CXCR2 showed synergistic effects with dexamethasone on outgrowth enhancement in microglia native OBSC, but it had no impact on spheroid outgrowth in the PLX5622-pretreated, microglia-depleted OBSC microenvironment. 

Preclinically, blocking the CXCR2-axis showed less tumor outgrowth in mouse experiments, but no advantage was seen in clinical trials [34,35]. Interestingly, in our model, blocking of the CXCL2-CXCR2-axis showed highly context-dependent effects depending on whether dexamethasone treatment and/or PLX5622 pretreatment were involved. We are aware that CXCL1-3 and CXCL5-8 contain an ELR motif showing high binding affinity for CXCR2 and that, additionally, in humans, IL-8 is one of the main ligands for CXCR2 [49]. The proteome profiler we used only included as a single CXCR2-binding ligand CXCL2. Consequently, we only did further studies on this ligand and the respective effector receptor CXCR2. CXCL3 or the other above-mentioned possible ligands were not included in the proteome profiler, nor did we check their secretion in our model. As therapies with CXCR2-blocking and microglia-depletion are considered therapeutical (yet still experimental) approaches in glioblastoma therapy, and dexamethasone is a very common symptomatic treatment agent in glioblastoma, we hereby produced meaningful results concerning further studies and clinical evaluations.

In our model, the pro- or anti-tumoral impact of microglia within the first days of interaction with tumor cells once more highlights why microglia-centered strategies against glioblastoma, such as depletion or re-education with agents like PLX5622, have failed in many studies [50].

The interrelations of dexamethasone, PLX5622-pretreatment (i.e., microglial depletion), and the CXCL2/CXCR2-axis underscore the difficulty to come up with treatment options that prove effective in various contexts. 

Our artificially created rat ex vivo OBSC model with rat S635 GBM cell line reflects the in vivo scenario only conditionally and holds major limitations with a number of possible confounding factors that are not controlled for:

- External stimuli that can induce general microglia- and tumor microenvironment-modulating effects;
- Sustained unspecific effects of the CSF1-R inhibitor PLX5622 on various cell types of the brain slice co-culture, which might generally minimize the validity of the conclusions from the model;
- Our evaluations of the cytokine signaling only allow for analyzing the general humoral fingerprint/general CXCL2 secretion—the exact secreting cells remain elusive;
- CXCL1-3 and CXCL5-8 contain an ELR motif showing high binding affinity for CXCR2; CXCL3 or the other above-mentioned possible ligands were not included in the proteome profiler, nor did we check their secretion in our model;
- Our exploratory study was conducted only on a single cell line—control for cell line dependencies is not given and, therefore, generalizability is very limited.

Even high-throughput studies (e.g., scRNA-Seq studies) do not take dexamethasone treatment into account, even though dexamethasone may cause significant changes on the cellular level within the tumor microenvironment. Further studies, especially a comparison between samples from dexamethasone-naïve and dexamethasone-treated patients, with regard to changes in tumor microenvironment and cytokine signaling, are necessary to contextualize and validate our findings from our model and our single rat glioblastoma cell line. Moreover, pre-clinical studies should involve dexamethasone co-treatment to check if the effects of the therapeutical effects are leveled or even reversed by dexamethasone that is routinely applied in the clinical setting. 

## 4. Materials and Methods

### 4.1. Cells

S635 cells and rat astrocytes were provided by Prof. Proescholdt (Department of Neurosurgery, University Regensburg, Regensburg, Germany). The S635 cell line was originally derived from a chemically induced brain tumor in Fischer 344 rats [51]. Rat astrocytes were obtained from newborn rats d6-14 by mechanically mincing the parenchyma and sieving through a 100 μm cell strainer (Falcon, 352360). Cells were seeded into a T75cm^2^ flask and one day after isolation medium was exchanged. Astrocytes were split at 90–95% confluency in a standard incubator at 37 °C and 5% CO_2_. Archiving in liquid nitrogen was performed and cells were thawed and seeded when needed.

Rat glioblastoma cell lines and astrocyte cells were cultivated in DMEM (Dulbecco’s Modified Eagle’s, Medium, NaHCO_3_ 3.7 g/L, D-Glucose 4.5 g/L, low endotoxin) supplemented with 10% FBS (Merck Biochrom, Berlin, Germany), 2 mM L-glutamine, and 100 U/mL penicillin/streptomycin (Merck Biochrom, Berlin, Germany) at 37 °C and 5% CO_2_ and split at a confluence of 90–95%.

### 4.2. Fluorescent Cell Labeling and Spheroid Formation

Cell lines were cultivated in serum-containing medium, were trypsinized (Trypsin/EDTA solution, Merck Biochrom, Berlin, Germany) and counted. 

Next, 1 × 10^6^ cells/mL in PBS (Sigma-Alrich, St. Louis, MO, USA) were incubated with 5 μL lipophilic dye DiD (CellBrite^®^ green #30021, Biotium, Fremont, CA, USA) for 30 min at 37 °C. Cells were washed twice in PBS, and then 4000 cells/well were seeded in a flat-bottom 96-well plate coated with 100 μL low melt agarose (Biozym Scientific, Oldendorf, Germany, 840100; 1% in PBS). Rat glioblastoma and astrocyte cells were cultivated in DMEM culture medium with FBS on agarose for 3–5 days until spheroid formation was completed, and then either put in U-bottom wells for spheroid migration or on the OBSC within the hippocampal area. 

### 4.3. Spheroid Migration Assay

For the spheroid migration, spheroids were transferred into agarose-free U-bottom 96-well plates (TPP, Trasadingen, Switzerland, #92097) and cultured in serum-free DMEM medium (Merck, Biochrom, Berlin, Germnay) for 7 days. Images were taken on day 0 and day 7 (4× magnification) with an inverted microscope (Olympus IX70, Olympus Life Science, Evident Europe, Hamburg, Germany) to be able to measure the cell-covered area within the U-bottom well. Images were evaluated and a concentrical increase of the area covered with cells was performed manually and in a blinded manner with ImageJ. Supernatants were collected and stored at −20 °C for baseline measurement of cytokine secretion of the cell line cells.

### 4.4. ATP-Assay

We used Cell Titer Glo Luminescent Cell Viability Assay (G7570, Promega, Madison, WI, USA). Cell Titer Glo Buffer and Cell Titer Glo Substrate were mixed until the substrate was completely dissolved in buffer. Per 96-Well plate well, we have cultured 4000 cells with or without dexamethasone treatment (0.1 µg/mL and 1 µg/mL) in serum-free DMEM medium (Merck, Biochrom, Berlin, Germany) for 7 days. Further, 100 µL/well Cell Titer Glo reagent was added per well, and put on a shaking plate for 12 min to induce cell lysis. After that, 150 µL of the supernatant was pipetted into a 96-well (F-bottom/chimney well, white lumitrac 600) plate and luminometric measurement was acquired with Varioskan flash (Thermofisher Scientific, Vantaa, Finland). 

### 4.5. Ex Vivo Rat Brain Slice Assay—Organotypic Rat Brain Slice Culture (OBSC)

#### OBSC Preparation

The brains of 6- to 14-day-old Fischer rat pups were isolated and the cerebellum was removed. Further, 350 μm thick coronal slices were cut with a vibratome (Leica VT1200 S, Leica Biosystems, Wetzlar, Germany), and two hippocampal slices were put on one 0.4-µm pore size filter each (Corning, REF 353090) in 6-well low evaporation plates (Corning, REF 353046) floating on brain slice medium. For 100 mL brain slice medium, 0.8 g MEM powder (Life Technologies, Paisley, UK, 11700-077), AquaDest 44.57 mL, HBSS 25 mL (Merck Biochrom, Berlin, Germany), 1.2 mL gluose 10%, 2 mL L-glutamine 200mM, 2 mL Penicillin, 0.05% (*v*/*v*), and Streptomycin, 25 mM mix, 100 µL ascorbic acid 4 mM, 50 µL media supplements (ITS, SITE, SPITE, Fatty Acid-Albumin Supplements, Sima-Aldrich, St. Louis, MO, USA), 580 µL bicarbonate 7.5%, and 500 µL Tris-base 1M were mixed and sterile-filtered, 25 mL 5% heat-inactivated horse serum (Life Technologies, 26050) was added, and eventually pH was adjusted to 7.4 with HCl [52,53].

Slices were cultured at 37 °C and 5% CO_2_. Medium was exchanged 24 h after culture initiation and then was refreshed in maximum 72 h intervals.

After optional pretreatment (see below) for up to 10 days, one 4000-cell spheroid per brain slice was manually put within the hippocampal area with a 200 µL pipette tip and a binocular microscope.

At day 0 and day 7, imaging was performed under the microscope (Olympus IX 70), and fluorescent intensity thresholding was performed using ImageJ (v1.53) in order to determine the extent of spheroid outgrowth objectively.

Supernatants were collected at d0, d3, and d6 and stored at −80 °C for further investigations. Representative viability checks with propidium iodide (PI) were performed 18 days after preparation of the OBSC. For this purpose, PI was added with a concentration of 1 mg/mL to the culture medium for at least 15 min. The evaluation was done under the microscope (Olympus IX 70).

### 4.6. PLX5622-Pretreatment of OBSC/Dexamethasone Treatment

For the PLX5622-pretreated condition, OBSC-slices were treated with 2 µM CSF1-R antagonist PLX5622 (MedChemExpress, HY-114153/CS-0077157, Monmouth Junction, NJ, USA), which was applied into the culture medium for 2 × 3 days. PLX5622 seems to be more efficient in microglia-depletion compared to other microglia-depleting systems (such as clodronate) and has a very specific, microglia-depleting effect, with only minor lasting effects on other cells [36,54]. With two days interim-treatment with culture medium, PLX5622 was washed out from the OBSC (see Figure 6, bottom). Native OBSC slices were cultured in parallel for the same period but without PLX5622-treatment and parallel exchanges of the culture medium. 

The 0.1 µg/mL dexamethasone dose was defined according to human pharmacokinetic peak plasma concentrations, which range 8.4+/−3.6 µg/L/1 mg dose [55] at established clinical doses in GBM patients ranging from 8 to 12 mg, and an excessive dexamethasone dose (power of ten) was tested (1 µg/mL).

### 4.7. Cytokine Screening Proteome Profiler

For the screening for differential signaling in 6 conditions (OBSC without spheroid +/− PLX5622-pretreatment, OBSC with spheroid +/− PLX5622-pretreatment, OBSC with dexamethasone +/− PLX5622-pretreatment), we performed a semi-quantitative membrane-based Proteome Profiler (XL rat Cytokine Profiler, Cat: DY994, R&D, Minneapolis, MN, USA) according to the manufacturer’s protocol. For the screening, we used pooled supernatants from d3 and d6 from 4 different replicates (pool from n = 8) in each of the above-mentioned conditions. Evaluation was performed with QuickSpots software (HLImage++, Western Vision Software, https://idealeyes.com/products/QuickSpots.php accessed on 22 August 2022). 

### 4.8. Enzyme-Linked Immunosorbent Assay (ELISA)

Enzyme-linked immunosorbent assay (Rat CXCL2/CINC-3 DuoSet ELISA, Cat: DY525, R&D, Minneapolis, MN, USA) was performed according to the manufacturer’s protocol. 

### 4.9. CXCL2/CXCR-Inhibitors

Danirixin (MedChemExpress, #HY19768/CS-5465), navarixin (MedChemExpress, #HY-10198/CS-0609), and, CXCL2 (Murine MIP-2, CXCL2; Pepro-Tech, #0516152 D1422) were applied with the culture medium in the respective concentrations.

### 4.10. Immunofluorescence Staining (GFAP, Iba1)

For GFAP-staining, we used an unconjugated, polyclonal anti-Glial Fibrillary Acidic Protein (GFAP) antibody (Z0334, DAKO, Würzburg, Germany). Cryosections (7 µm, obtained from Leica, CM 1950; on Superfrost Plus, Thermo Fisher, Braunschweig, Germany) were thawed, air-dried, and fixated for 10 min in 4% paraformaldehyde at room temperature, washed with PBS for 3 × 10 min, and then blocked with blocking solution (3% dk Serum/PBS-0.1% Triton × 100). Incubation with the primary antibody was done overnight (1:200) at 4 °C and washed afterwards 3 × 10 min in PBS. For GFAP-staining, we used the secondary antibody conjugated with Rhodamin Red-X (1:100 in blocking solution, donkey anti-rabbit, 711-295-152 dianova, Hamburg, Germany) and incubated for 2 h at room temperature. 

For the microglial staining, we used unconjugated Iba1 polyclonal antibody (PA5-27436, ThermoFisher Scientific). Cryosections were thawed, air-dried, and then fixated for 15 min in 4% paraformaldehyde, were washed 3 × 10 min in PBS, and afterwards permeabilized in TritonX (0.1% in PBS, Sigma Aldrich) for 10 min and then washed again 2 × 10 min in PBS. Blocking was performed for 1 h with 2% BSA (bovine serum albumin, PAA, Pasching, Austria). The tissue sections were incubated with Iba1 polyclonal antibody (1:200) overnight at 4 °C. Slides were washed 3 × 10 min in PBS before the secondary antibody conjugated with Rhodamin Red-X (1:100 in 0.1% BSA, donkey anti-rabbit, 711-295-152 dianova, Hamburg, Germany) was applied for 2 h at room temperature. 

After secondary antibody incubation, another washing step (2 × 10 min in PBS) was done, and the nuclei were stained with Hoechst 33345 (1:20,000, Thermo Fisher Scientific) with an incubation time of 10 min at room temperature. After another washing step, DAPCO (1,4-Diazabicyclo[2.2.2]octan) was used for coverslipping (Menzel Gläser, Thermo Fisher, Braunschweig, Germany).

Whole Slide Images were acquired with Nikon Eclipse Ti2 (Amstelveen, Netherlands) in multichannel mode (DAPI-, Cy3-, GFP-channels) at 40× magnification and MMI CellTools Software version 6.0 (Molecular Machines & Industries, Eching, Germany). Data sets were processed with ImageJ.

### 4.11. Statistical Analysis

Experiments were performed in at least three independent replicates and, whenever possible, analysis was performed in a blinded manner. Statistics were calculated and statistical graphs were generated with GraphPad Prism v.9.5.1 (GraphPad Software Inc., Boston, MA, USA). Parametric testing was done with the student’s *t*-test, and comparisons between multiple groups were done using one-way ANOVA or with multiple parameters with two-way ANOVA. * *p* < 0.05; ** *p* < 0.01; *** *p* < 0.001; **** *p* < 0.0001; “ns” implies a non-significant *p* value. All figures are presented as means and standard deviation.

## 5. Conclusions

Even though we used a very controlled model with only three variables (native/PLX-pretreatment, dexamethasone treatment, CXCR2-inhibition), the effect on S635 GBM cell line outgrowth is highly context-dependent and interactive due to interrelations and/or synergistic effects of the factors (humoral, cellular, and drug treatment). When CXCR2 is inhibited, native OBSC TME seems to mediate tumor-permissive effects, and even tumor-promoting effects are produced in combination with dexamethasone treatment—this phenomenon is contrary or missing when PLX5622-pretreatment of the OBSC was performed. Breaking down GBM complexity to a simplistic and mechanistic model with only three variables still generates high context-dependencies, which point at an even more complex setting within the human peritumoral area of the GBM that has to be further evaluated.

## Data Availability

The data sets generated and analyzed during the current study are available from the corresponding author upon reasonable request.
